# Knocking Down the Expression of GMPase Gene *OsVTC1-1* Decreases Salt Tolerance of Rice at Seedling and Reproductive Stages

**DOI:** 10.1371/journal.pone.0168650

**Published:** 2016-12-19

**Authors:** Hua Qin, Yayun Wang, Juan Wang, Hai Liu, Hui Zhao, Zaian Deng, Zhili Zhang, Rongfeng Huang, Zhijin Zhang

**Affiliations:** 1 Biotechnology Research Institute, Chinese Academy of Agricultural Sciences, Beijing, China; 2 National Key Facility of Crop Gene Resources and Genetic Improvement, Beijing, China; 3 Department of Biology, University of Virginia, Charlottesville, VA, United States of America; 4 Hainan Academy of Agricultural Sciences, Haikou, China; University of Western Sydney, AUSTRALIA

## Abstract

Salinity is a severe environmental stress that greatly impairs production of crops worldwide. Previous studies have shown that GMPase plays an important role in tolerance of plants to salt stress at vegetative stage. However, the function of GMPase in plant responses to salt stress at reproductive stage remains unclear. Studies have shown that heterologous expression of rice GMPase *OsVTC1-1* enhanced salt tolerance of tobacco seedlings, but the native role of *OsVTC1-1* in salt stress tolerance of rice is unknown. To illustrate the native function of GMPase in response of rice to salt stress, *OsVTC1-1* expression was suppressed using RNAi-mediated gene silencing. Suppressing *OsVTC1-1* expression obviously decreased salt tolerance of rice varieties at vegetative stage. Intriguingly, grain yield of *OsVTC1-1* RNAi rice was also significantly reduced under salt stress, indicating that *OsVTC1-1* plays an important role in salt tolerance of rice at both seedling and reproductive stages. *OsVTC1-1* RNAi rice accumulated more ROS under salt stress, and supplying exogenous ascorbic acid restored salt tolerance of *OsVTC1-1* RNAi lines, suggesting that *OsVTC1-1* is involved in salt tolerance of rice through the biosynthesis regulation of ascorbic acid. Altogether, results of present study showed that rice GMPase gene *OsVTC1-1* plays a critical role in salt tolerance of rice at both vegetative and reproductive stages through AsA scavenging of excess ROS.

## Introduction

GDP-d-mannose pyrophosphorylase (EC 2.7.7.13, d-mannose-1-phosphate guanylyltransferase, GMPase) catalyzes the conversion of d-mannose-1-P to GDP-d-mannose in mammals, plants, yeasts and bacteria [1]. In plants, GDP-d-mannose is used to synthesize not only structural carbohydrates but also ascorbic acid (AsA) [2–4]. Reflecting multiple metabolite pathways involving GDP-d-mannose, GMPase is of great importance in developmental processes and responses to environmental stresses [5–10].

GMPase acts as a key factor in cell division, flowering, and senescence of plants [8, 9, 11–13]. *cyt1*, a knockout mutant of GMPase in *Arabidopsis*, is embryonic lethal. Embryonic development in *cyt1* is arrested at an early stage, resulting in plant tissues with severely abnormal cell walls, reflecting deficiency of GMPase [14, 15]. *vtc1* is a knockdown allelic mutant of *cyt1* with reduced GMPase activity [5, 16] and GDP-mannose synthesis, resulting in the deficiency of cell wall carbohydrates and protein N-glycosylation [8, 12, 17]. Inhibiting GMPase expression in tobacco results in a phenotype with smaller leaves and early flowering [9].

GMPase also plays various functions in the response of plants to biotic and abiotic stresses [9, 18–20]. Mutant *vtc1-1* showed pathogen resistance through H_2_O_2_, salicylic acid, and gibberellic acid signaling pathways to regulate the expression of defense genes [21–24]. Barth [22] and Pavet [23] showed that *vtc1-1* can inhibite the growth of virulent bacterial pathogen *Pseudomonas syringae* pv. *maculicola* ES4326 and virulent *Oomycete Hyaloperonospora parasitica* pv. *Noco* by enhancing the transcript and protein levels of pathogenesis-related (PR) proteins. Results of latter study suggest that the pathogen resistance mechanism of *vtc1-1* probably reflects the accumulation of SA and transcripts of PR genes through H_2_O_2_ buildup [24]. In addition, GMPase is also involved in responses to abiotic stresses. For example, in tobacco, overexpression of GMPase gene increases the tolerance to low and high temperature stresses. In contrast, inhibiting GMPase expression decreased the tolerance of tobacco to temperature stresses [9, 25]. *Arabidopsis* mutant *vtc1* shows impaired tolerance to salt stress, reflecting a dramatic decrease in the production of antioxidant AsA and deficiency in scavenging reactive oxygen species (ROS) under salt stress [19]. *vtc1* allelic mutant, *soz1*, is also deficient in AsA and hypersensitive to both sulfur dioxide and ultraviolet B irradiation [16]. Overexpression of tomato GMPase, SlGMP3, enhances tolerance of tomato to oxidative stress [13]. These studies showed that GMPase plays an important role in the responses to environmental stresses [18, 26, 27]. However, most early studies are focused on the function of GMPases in salt tolerance of plants at vegetative stage, the role of GMPases in salt stress at reproductive stage remains unclear.

Previous study showed that there are three GMPase homologous genes, named as OsVTC1-1, OsVTC1-3 and OsVTC1-8 in rice. OsVTC1-1 and OsVTC1-3 are involved in AsA biosynthesis of rice, and OsVTC1-1 is responsible for most production of AsA in rice leaf [28]. In addition, results from Kumar [29] showed that heterologous overexpression of *OsVTC1-1* improved the tolerance of yeast and tobacco to salt stress, indicating that *OsVTC1-1* may play an important role in the response of rice to salt stress, but the native role of GMPases in response of rice to salt stress is not well understood. To further study the function of *OsVTC1-1* in the response to salt stress in rice, we knocked down the expression of *OsVTC1-1* in rice by RNA interference. In present study, we showed that *OsVTC1-1* plays a critical role in the response of rice to salt stress not only at seedling stage but also at reproductive stage.

## Materials and methods

### Plant materials and growth conditions

Seeds of different rice varieties were germinated in a greenhouse at 30°C (16-hour light) and 22°C (8-hour dark). *Japonica* cultivar ZH17 (*Oryza sativa* L. ssp. *japonica* cv. Zhonghua 17) was used as wild-type (WT) sample in the present study, except where explicitly indicated. *Arabidopsis* seeds were maintained at 4°C for 4 days and subsequently germinated and grown in an incubator with 14-hour lighting (22°C) and 10-hour darkness (18°C). To examine the role of *OsVTC1-1* in the tolerance of rice to salt stress at reproductive stage, ZH17 and *OsVTC1-1* RNAi (RI) rice seedlings were transferred into a small concrete pool (length: 60 cm, width: 60 cm, and depth: 80 cm).

### Genetic transformation of *Arabidopsis* and rice

Full-length ORF of *OsVTC1-1* was cloned into the plant expression vector pCAMBIA 1307 using the *Xba* I and *Sal* I sites, and subsequently transformed into *vtc1-1* using the floral dip method [30, 31]. The transgenic *OsVTC1-1* overexpression lines were selected using hygromycin and confirmed through western blotting. Homozygous T3 transgenic lines were used in the present study, as previously described [28].

*OsVTC1-1* knockdown rice plants were established using RNA interference (RNAi) technology. Briefly, forward and reverse *OsVTC1-1* 3’UTR specific sequences were cloned into pUCCRNAi vector using endonuclease. The forward and reverse *OsVTC1-1* DNA fragments in pUCCRNAi vector were further cloned into plant vector pCAMBIA2300 and subsequently introduced into *Zhonghua17* rice using *Agrobacterium*-mediated transformation. The transformed plants were selected using G418 and confirmed through PCR, as previously described [28]. The homozygous T3 transgenic rice lines RI1-1, RI1-2 and RI1-3 were used in present study.

### The production of hybrid rice

To limit the expression of *OsVTC1-1* in salt-tolerant local rice varieties Ningjing 16 (NJ16) and Zhongdao 13 (ZD13), we generated the hybrid rice NJ16/RI1-3 and ZD13/RI1-3 using *OsVTC1-1* RNAi line RI1-3 as male parent and lines NJ16 and ZD13 as female parents, respectively. The hybrid plant lines NJ16/ZH17 and ZD13/ZH17 derived from the hybrid of ZH17 (male parent) with NJ16 and ZD13 (female parent), respectively, were used as controls. F1 plants of hybrid rice were selected using G418 (100μg/ml) for two weeks and subsequently used to analyze the transcript level of *OsVTC1-1* and characterize the tolerance to salt stress.

### Quantitative real-time PCR (qPCR)

To examine the expression of *OsVTC1-1* gene under different stress treatments, four-week-old ZH17 seedlings were sprayed with 200 mM NaCl solution, 10% (w/v) PEG6000 solution or 100 mM MV (Methyl Viologen), harvested at different time points and subsequently stored in liquid nitrogen for RNA extraction. Four-week-old seedlings were used to isolate total RNA from F1 plants NJ16/RI1-3 and ZD13/RI1-3. Total RNA was extracted using TRIzol solution (Tiangen, Beijing, China). Subsequently, approximately 2 μg of total RNA was reverse transcribed using oligo (dT) primers and M-MLV reverse transcriptase according to the manufacturer’s instructions (Toyobo, Osaka, Japan). qPCR was performed using 2X SYBR Green mix (Takara, Cat No. 330523) and iQ5 according to the manufacturer’s instructions (Bio-Rad iQ5). Primers 5’-GACCTTGCTGGGCGTGAT-3’ and 5’-GTCATAGTCCAGGGCGATGT-3’ were used for *OsActin1*, and primers 5’-GTCATGTGAACTAACCCTCC-3’ and 5’-GAGTTTCTTCTGGTCCTCTTG-3’ were used for *OsVTC1-1*. For analyzing the expression character of *OsVTC1-1* under abiotic stresses, the transcript level of *OsVTC1-1* under control conditions (0 h) was assigned as 1, and what results shows in figure was relative expression levels of *OsVTC1-1* at others times relative to 0 h. For analyzing the expression character of *OsVTC1-1* in F1 plants, the transcript level of *OsVTC1-1* under ZH17 was assigned as 1, and what results shows in figure was relative expression levels of *OsVTC1-1* to that in ZH17. qPCR reactions were performed in biological triplicate (data of each replicate from the average of three parallel samples)for each individual line, and threshold cycle values were quantified using relative quantification method. The expression of *OsActin1* gene was used as internal standard, and the relative expression values of *OsVTC1-1* were calculated using comparative Ct method [32].

### Analysis of salt stress tolerance

To examine salt tolerance, wild-type, mutant (*vtc1-1*) and transgenic seven-day-old *Arabidopsis* seedlings were cultured on Murashige and Skoog (MS) plates with or without 150 mM NaCl for ten days [19, 28]. To examine the tolerance of *OsVTC1-1* RI plants to salt stress, two-week-old *OsVTC1-1* RI seedlings were treated with 150 mM NaCl solution for two weeks, followed by watering without NaCl for an additional ten days. Subsequently, the percentage survival rate of rice seedlings was determined (plants with green leaves represented surviving seedlings). To evaluate the role of *OsVTC1-1* RI in the response of rice to salt stress at reproductive stage, two-week-old seedlings of ZH17 and *OsVTC1-1* RNAi (RI) plants were transferred into a small concrete pool, grown for another four weeks under normal growth conditions, and subsequently watered with 100 mM NaCl until harvest. To examine the salt-tolerant of different rice landraces, F1 hybrid seeds of NJ16/RI1-3 and ZD13/RI1-3 were germinated and selected on G418 (100μg/ml) for two weeks and subsequently planted and grown in soil for two weeks prior to watering with 150 mM NaCl for another ten days. Moreover, seedlings were watered without NaCl for ten days; subsequently, the percentage survival rates were calculated. For treatments with both NaCl and AsA, ZH17 and *OsVTC1-1* RI seeds were cultured on MS medium (without sucrose), germinated and grown at 30°C for four days. Then seedlings were transferred to MS medium (without sucrose) containing 150 mM NaCl and cultivated for another five days with or without 10μM exogenous AsA. Subsequently, phenotypes were observed.

### AsA content

To measure the AsA levels, four-week-old rice seedlings were harvested and stored in liquid nitrogen. A 0.2-g sample (fresh weight) of seedlings was ground to fine powder in liquid nitrogen with mortar and pestle. The extraction and measurement of AsA were performed according to Wang et al. [33].

### MDA and chlorophyll contents

To analyze malondialdehyde (MDA) and chlorophyll contents under salt stress, two-week-old rice seedlings were grown in soil and watered with or without 150 mM NaCl for ten days. To measure MDA contents, protoplasts were extracted from 0.1 g of leaf tissue in 1 ml of 0.1% (w/v) trichloroacetic acid (TCA), and the mixture was boiled at 95°C for 15 min. Subsequently, 4 ml of 20% TCA containing 0.5% thiobarbituric acid (TBA) was added, and the mixture was immediately cooled on ice. MDA content was determined after measuring the optical density at 532 and 600 nm, according to the methods of Zhang et al. [34].

To detect chlorophyll content, 0.1 g of leaf tissue was ground into powder, mixed with 100% dimethyl formamide at a ratio of 1:20 (w:v) and subsequently centrifuged at 10,000 g for 5 min. Chlorophyll content was determined after measuring the optical density at 664 and 647 nm. Total chlorophyll level (chlorophyll a + chlorophyll b) was determined according to Aono et al. [35].

### O_2_^-^ staining

To analyze O_2_^.-^ content under salt stress, two-week-old rice seedlings were grown in soil and watered with or without 150 mM NaCl for two days. O_2_^.-^ polymerizes nitrotetrazolium blue chloride (NBT) into a blue deposit. To analyze O_2_^.-^ content, rice leaves were incubated in 25 mM HEPES buffer (pH 7.6) containing 1% NBT in dark at 25°C for 12 hours. Next, these leaves were destained with 80% ethanol (v/v) until chlorophyll was removed and clear blue deposits were observed.

## Results

### The expression of *OsVTC1-1* was induced by abiotic stresses

**Fig 1:**
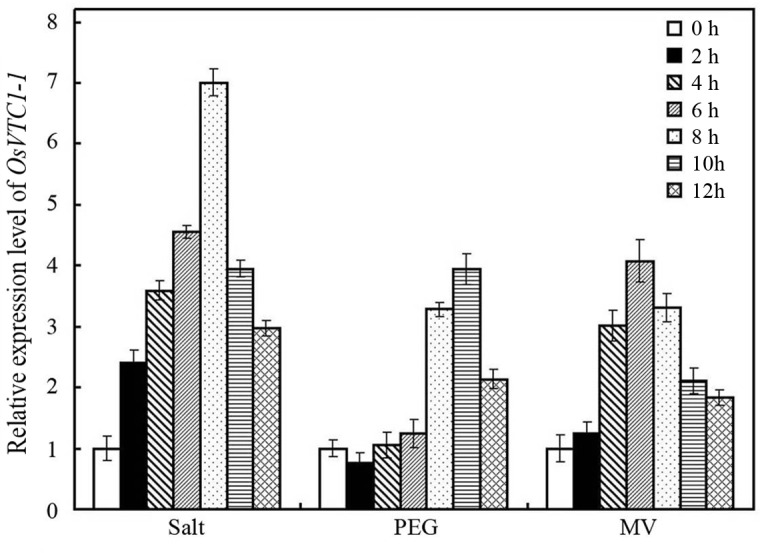
The expression of *OsVTC1-1* in plant responses to abiotic stresses. To examine the expression patterns of *OsVTC1-1* in plant responses to salt, PEG or MV, the transcript level of *OsVTC1-1* was analyzed by Q-PCR. After normalizing to internal control (*Actin*), the transcript level of *OsVTC1-1* under control conditions (0 h) was assigned as 1, and this figure shows the expression level of *OsVTC1-1* at others times relative to 0 h. The experiments were repeated three times.

To illustrate the role of *OsVTC1-1* in the response of rice to salt stress, we analyzed the expression patterns of *OsVTC1-1* under abiotic stresses. The qPCR results showed that, similar to the results reported by Kumar [29], the expression *OsVTC1-1* is induced through salt (NaCl) treatment (Fig 1). In addition, the expression of *OsVTC1-1* was also induced after oxidative stress treatment (Methyl Viologen, MV) and osmotic stress treatment (PEG 6000), although gene expression patterns showed some difference under these treatments (Fig 1). The expression of *OsVTC1-1* was rapidly induced at 2 hours after NaCl treatment and peaked at 8 hours. In contrast, the expression of *OsVTC1-1* was slightly induced at 8 hours after PEG 6000 treatment and peaked at 10 hours (Fig 1). The expression of *OsVTC1-1* was induced through MV within 4 hours and peaked at 6 hours (Fig 1).

### *OsVTC1-1* plays an important role in salt tolerance of rice seedlings

**Fig 2:**
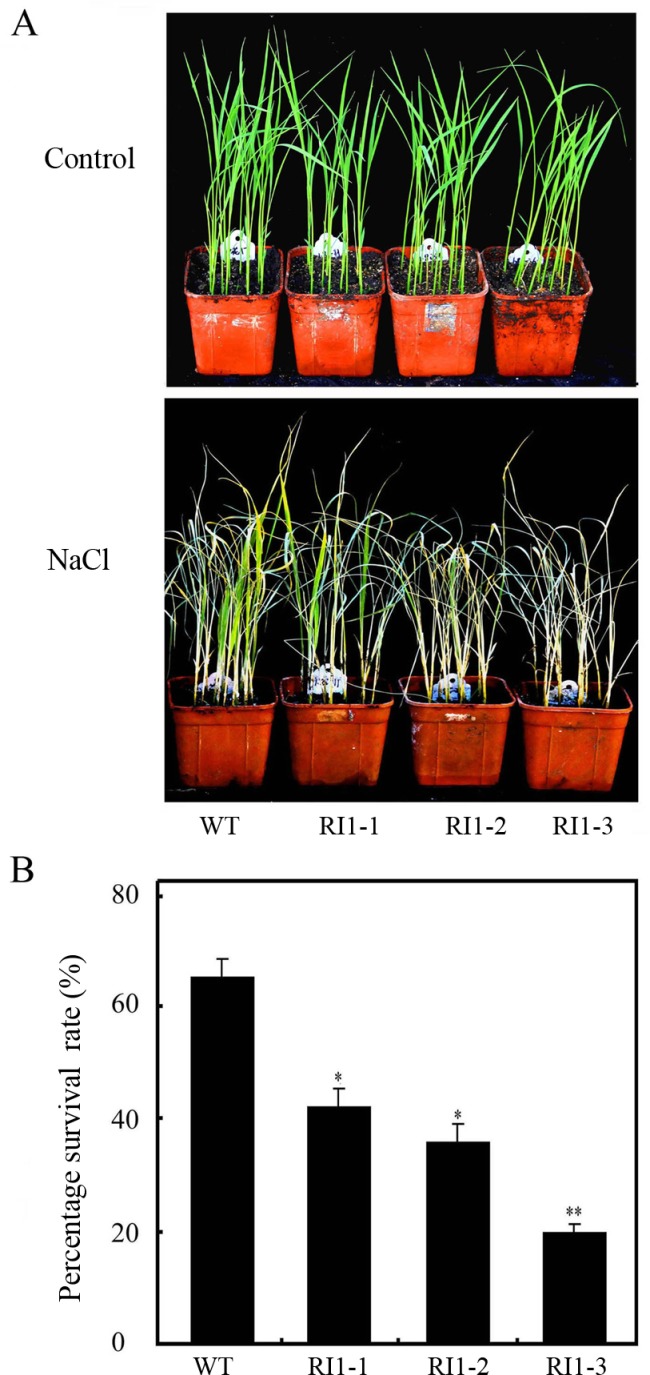
Decreasing the expression of *OsVTC1-1* impairs the tolerance of rice seedlings to salt stress. (A), phenotypes of *OsVTC1-1* RI lines under salt stress. Control indicates that rice seedlings were grown under normal conditions, and NaCl indicates that seedlings were treated with 150 mM NaCl aqueous solution. (B), the percentage survival rates (Percentage is the ratio of number of plants with green new leaves to number of total plants) of *OsVTC1-1* RI plants after salt treatment in (A). WT represents ZH17 rice variety; RI1-1, RI1-2 and RI1-3 indicate independent RNA interference lines of *OsVTC1-1* in ZH17 background, respectively. About 50~60 seedlings were uses in each experiment. The bars represent SE (±) of three independent assays, and the asterisk indicates results significantly different from WT (**P<0.01 and *P<0.05). Significance was evaluated using the *t*-test.

To examine the native function of *OsVTC1-1* in the response of rice to salt stress, we disrupted OsVTC1-1 activity after knocking down the expression of *OsVTC1-1* using RNA interference (RI) technology. Previous study showed that the expression of *OsVTC1-1* is significantly decreased in *OsVTC1-1* RI lines, but expressions of its homologous genes *OsVTC1-3* and *OsVTC1-8* are not obviously interfered [28]. Rice is sensitive to salt stress at seedling and reproductive stages [36]. First, we treated *OsVTC1-1* RI seedlings with salt (NaCl) to analyze the native function of *OsVTC1-1* in the response of rice to salt stress at seedling stage. When treated with 150 mM NaCl for two weeks, *OsVTC1-1* RI transgenic seedlings exhibited obviously decreased tolerance to salt stress (Fig 2A). After resuming cultivation for ten days, the percentage survival rate of *OsVTC1-1* RI lines also significantly decreased (Fig 2B). The percentage survival rate of wild-type rice seedlings was 64.8%, whereas that of *OsVTC1-1* RI1-3, which had 15% less *OsVTC1-1* transcript levels compared with wild-type, was approximately 20% [28]. This result indicated that *OsVTC1-1* plays a critical role in the response of rice to salt stress at seedling stage.

**Fig 3:**
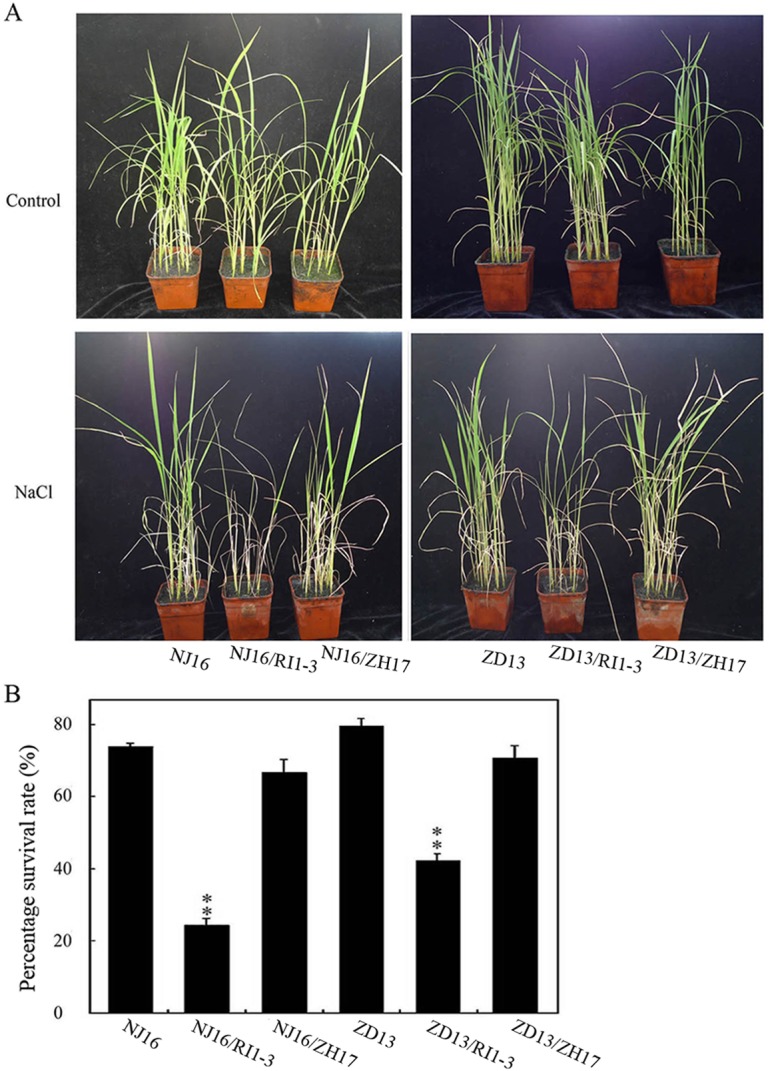
*OsVTC1-1* plays an important role in the response of salt-tolerant rice varieties. (A), phenotypes of F1 hybrid rice plants under salt stress. NJ16 represents salt-tolerant rice landrace Ningjing16; NJ16/RI1-3 represents F1 hybrid of Ningjing16 with *OsVTC1-1* RNA interference line RI1-3; NJ16/ZH17 represents F1 plants of Ningjing16 and Zhonghua17, which were used as controls. ZD13 indicates the salt-tolerant rice landrace Zhongdao13; ZD13/RI1-3 indicates F1 plants of Zhongdao13 and *OsVTC1-1* RI1-3; ZD13/ZH17 indicates F1 plants of Zhongdao13 and Zhonghua17, which were used as controls. Ningjing16 and Zhongdao13 were used as the female parents. Control indicates that plants were grown under normal conditions, and NaCl indicates that four-week rice seedlings were treated with 150 mM NaCl solution for another ten days. (B), percentage survival rates (Percentage is the ratio of number of plants with green new leaves to number of total plants) of F1 hybrid rice seedlings after salt stress treatment in (A). About 50~60 seedlings were uses in each experiment. The bars represent SD (±) of two independent assays, and the asterisk indicates results significantly different from control (**P<0.01 and *P<0.05). Significance was evaluated using the *t*-test.

To further illustrate the role of *OsVTC1-1* in the salt tolerance of rice, two salt-tolerant local varieties, DJ16 and ZD13, were selected to further examine the native function of *OsVTC1-1* in the response of rice to salt stress (S1 Fig). We utilized NJ16 and ZD13 to generate hybrids with *OsVTC1-1* RI1-3 plants (NJ16/RI1-3 and ZD13/RI1-3) and decrease the expression of *OsVTC1-1* in NJ16 and ZD13, respectively. The plants NJ16/ZH17 and ZD13/ZH17, produced from the hybridization of ZH17 with NJ16 and ZD13, respectively, were used as controls. Expression level of *OsVTC1-1* and synthesis of AsA were significantly suppressed in the F1 NJ16/RI1-3 and ZD13/RI1-3 hybrid plants (S2 Fig). We treated four-week-old F1 hybrid rice lines with 150 mM NaCl for ten days and subsequently resumed cultivation for an additional ten days. Results showed that salt tolerance of NJ16/RI1-3 and ZD13/RI1-3 plants was obviously decreased (Fig 3). After treatment with 150 mM NaCl, the percentage survival rates of NJ17/RI1-3 and ZD13/RI1-3 hybrids were 26.3% and 47.2%, respectively. In contrast, the percentage survival rates of NJ17/ZH17 and ZD13/ZH17 control plants after salt treatment were approximately 63.1% and 72.2%, respectively (Fig 3B). Above results showed that *OsVTC1-1* is a general factor for examining salt tolerance of rice at seedling stage.

### Downregulating *OsVTC1-1* expression reduces grain production in rice

**Fig 4:**
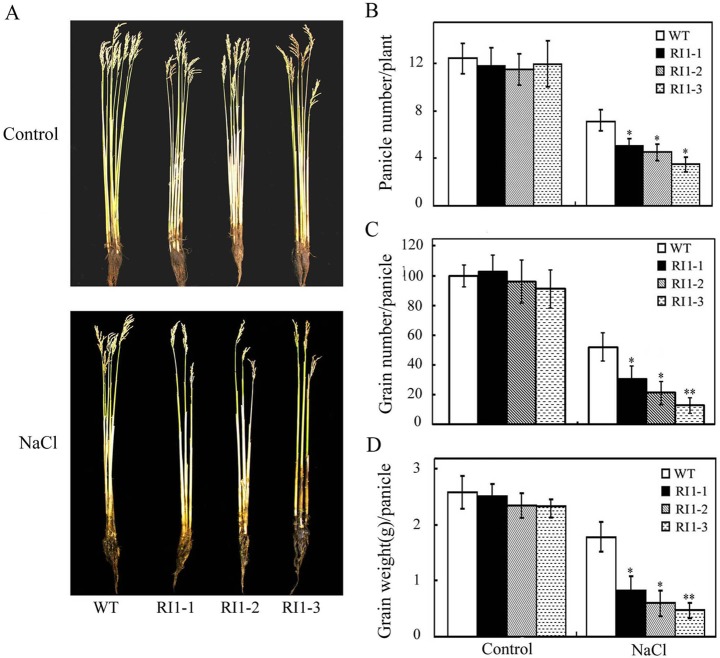
*OsVTC1-1* RI lines show reduced grain production under salt stress. (A), phenotypes of *OsVTC1-1* RI lines grown under salt stress at harvest stage. (B), panicle number of *OsVTC1-1* RI plants with or without salt treatment. (C), grain number of the panicle of *OsVTC1-1* RI plants with or without salt treatment. (D), production of the panicle of *OsVTC1-1* RI plants with or without salt treatment. Control indicates that plants were grown under normal conditions, and NaCl indicates that plants were treated with 100 mM NaCl. WT represents ZH17; RI1-1, RI1-2 and RI1-3 indicate different RI lines of *OsVTC1-1* in ZH17 background. About 40~50 plants were uses in each experiment. Bars represent SE (±) of three independent assays, and the asterisk indicates that the results were significantly different from WT (**P<0.01 and *P<0.05). Significance was evaluated using the *t*-test.

Rice yield also is severely decreased by salt stress at reproductive stage [36, 37]. To further illustrate the role of *OsVTC1-1* in the tolerance of rice to salt stress, we analyzed the effect of *OsVTC1-1* on rice grain production under high salt condition. Six-week-old wild-type and RI transgenic plants growing in soil were watered with 100 mM NaCl until harvest. Results showed that inhibiting the expression of *OsVTC1-1* clearly reduced grain production of transgenic rice under salt treatment (Fig 4A). Under high salt conditions, *OsVTC1-1* RI lines produced less grains compared with control lines, reflecting fewer panicles and less grain number per panicle compared with control plants (Fig 4B, 4C and 4D). These results further indicate that OsVTC1-1 also plays a key role in the tolerance of rice to salt stress at reproductive stage.

### OsVTC1-1 controls salt tolerance of rice by controlling AsA production

**Fig 5:**
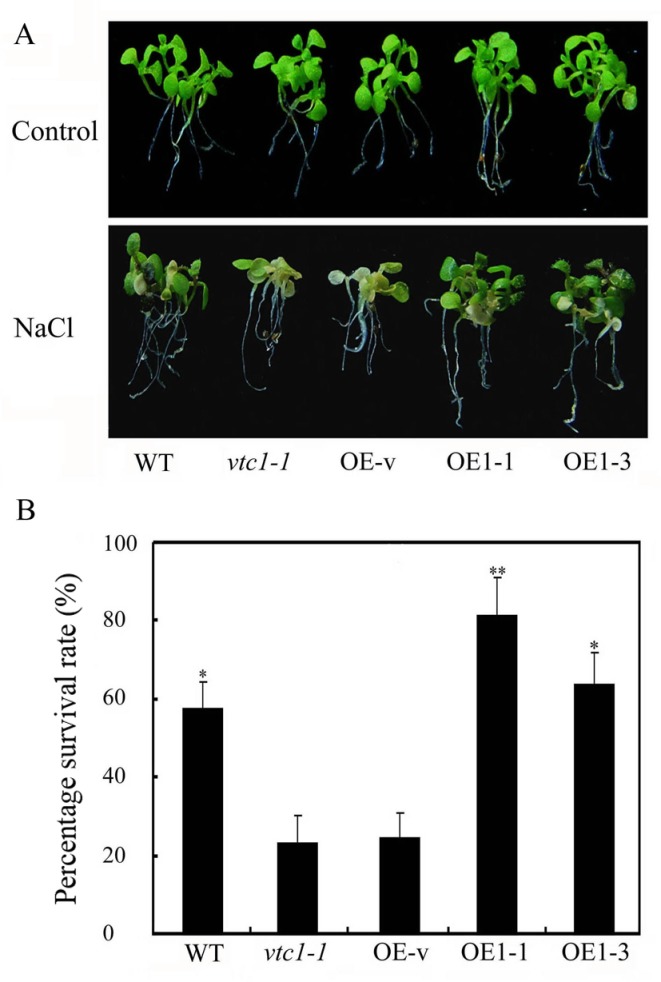
Overexpression of *OsVTC1-1* enhances the tolerance of *Arabidopsis* to salt stress. (A), phenotypes of *OsVTC1-1* overexpressing *Arabidopsis* under salt stress. Control indicates that plants were grown under normal conditions, and NaCl indicates that *Arabidopsis* seedlings were treated with 150 mM NaCl for ten days. (B), the percentage survival rates of *OsVTC1-1* overexpressing lines after salt treatment in (A). OE-v represents *vtc1-1* plants transformed with the pCAMBIA1307 blank vector. OE1-1 and OE1-3 indicate different overexpression lines of *OsVTC1-1* in *vtc1-1* background. WT represents wild-type Col-0 *Arabidopsis*. About 70~80 seedlings were uses in each experiment. The bars represent SE (±) of three independent assays, and the asterisk indicates results significantly different from *vtc1-1* (**P<0.01 and *P<0.05). Significance was evaluated using the *t*-test.

*Arabidopsis* mutant *vtc1-1* which only has approximately one-fourth AsA content of control plants impairs salt tolerance [19]. Transgenic *Arabidopsis* overexpressing *OsVTC1-1* (*vtc1-1* background [28]) restored AsA synthesis and recovered salt tolerance (Fig 5A and 5B), suggesting that OsVTC1-1 might be involved in the response of rice to salt through the regulation of AsA synthesis.

**Fig 6:**
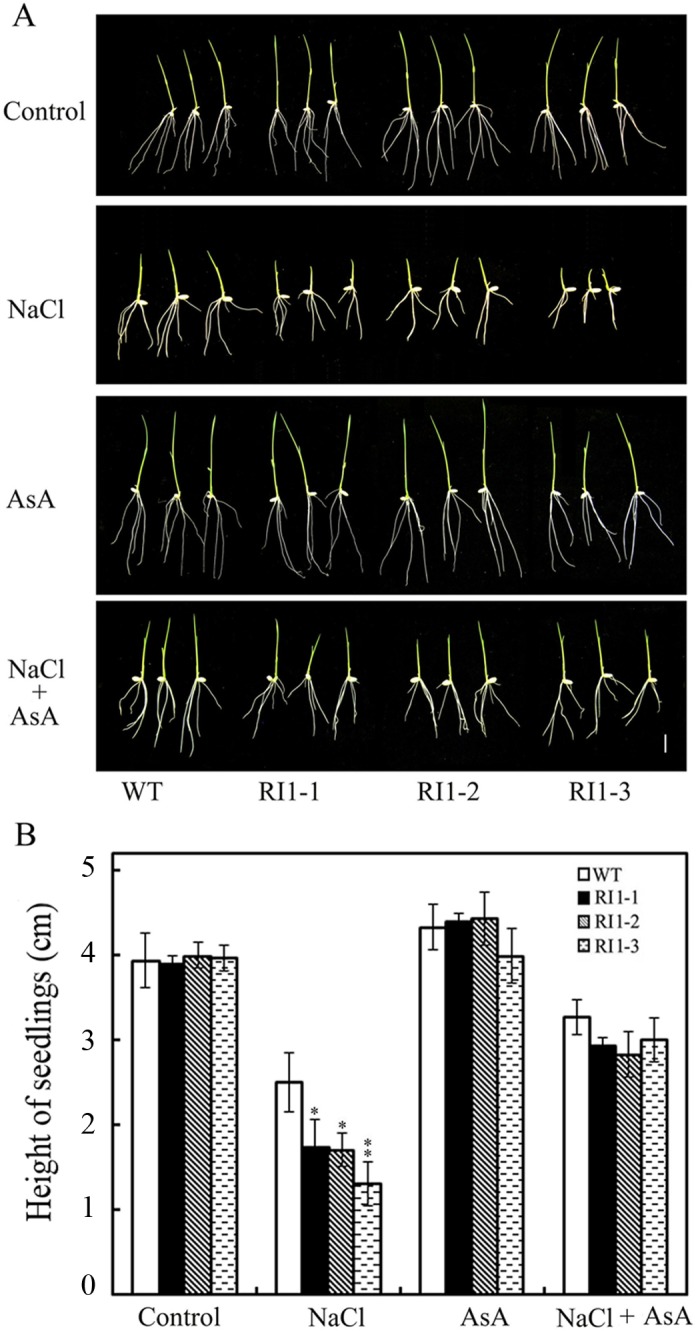
Exogenous AsA rescues the tolerance of *OsVTC1-1* RI plants to salt stress. (A), phenotypes of *OsVTC1-1* RI lines grown on MS medium with or without supplying exogenous AsA under salt treatment. (B), height of OsVTC1-1 RI lines seedlings grown on MS medium with or without supplying exogenous AsA under salt treatment. Control indicates that rice seedlings were grown on MS medium; NaCl indicates that rice seedlings were grown on MS medium with 150 mM NaCl; AsA indicates that rice seedlings were grown on MS medium with 10 μM AsA; and NaCl+ AsA represents rice seedlings grown on MS medium with 150 mM NaCl and 10 μM AsA. Above assays were repeated three times. About 50~60 seedlings were uses in each experiment. The bars represent SE (±). The asterisk indicates results significantly different from WT (**P<0.01 and *P<0.05). Significance was evaluated using the t-test.

Studies have demonstrated that the synthesis of AsA plays important roles in the response of plants to salt stress [25, 38–40]. To further analyze the role of AsA in *OsVTC1-1*-mediated enhancement of salt tolerance in rice, we examined the effect of AsA on salt tolerance of *OsVTC1-1* RI seedlings with supplying exogenous AsA. Result showed that exogenous AsA restored the tolerance of *OsVTC1-1* RI rice seedlings to salt stress (Fig 6). After supplying exogenous AsA, the growth of *OsVTC1-1* RI shoots was similar to that of wild-type (Fig 6B). This finding indicated that OsVTC1-1 is involved in response of rice to salt stress through the regulation of AsA biosynthesis.

### OsVTC1-1 is involved in ROS scavenging in rice under salt stress

**Fig 7:**
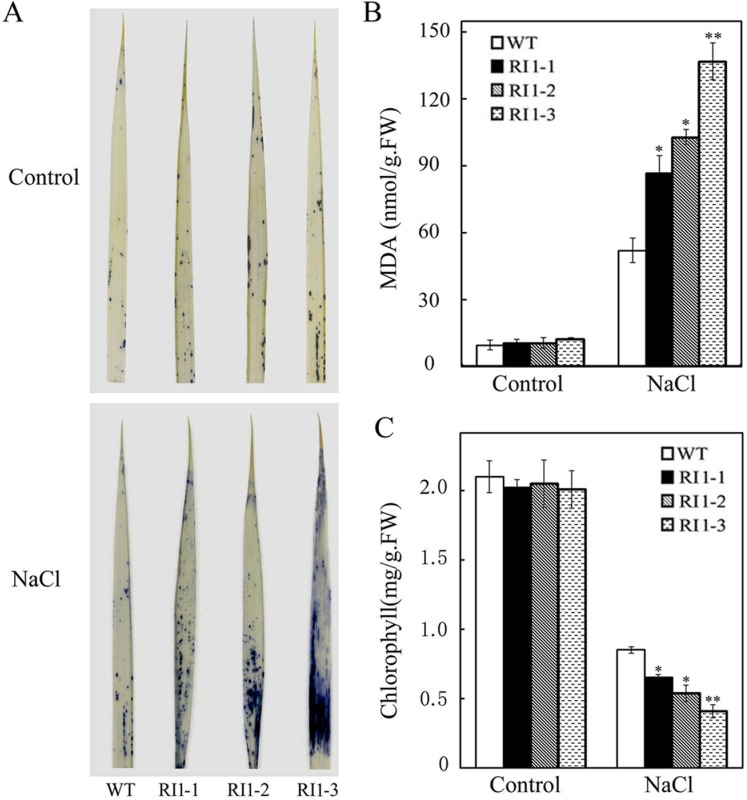
Downregulating expression of *OsVTC1-1* impairs the capacity of rice to scavenge ROS under salt stress. (A), O2^.-^ content of leaves in ZH17 (WT) and *OsVTC1-1* RI lines with or without salt treatment. O2^.-^ content was determined through histological staining with NBT. (B), MDA content of WT and *OsVTC1-1* RI lines with or without salt treatment. (C), chlorophyll content of WT and *OsVTC1-1* RI lines with or without salt treatment. Above assays were repeated three times. About 50~60 seedlings were uses in each experiment. The bars represent SE (±). The asterisk indicates results significantly different from WT (**P<0.01 and *P<0.05). Significance was evaluated using the *t*-test.

Environmental stresses, such as drought, salinity or disease, potentially harm plants through accumulation of ROS within plant cells [41, 42]. As the most abundant antioxidant in plants, AsA plays an important role in ROS scavenging to protect plants against ROS-induced damage under salt stress [25, 38, 39]. To examine the mechanism of *OsVTC1-1* in response to salt stress, we analyzed ROS (O_2_^-^) content in leaves of *OsVTC1-1* RI plants grown under salt stress. The data showed that the RI plants accumulated much more O_2_^-^ in the leaves compared with wild-type plants (Fig 7A), indicating that inhibiting the function of OsVTC1-1 reduces ROS scavenging in rice under salt stress. The accumulation of ROS damages cellular proteins, enzymes and lipids. Malondialdehyde (MDA) is an important product of lipid peroxidation, representing the degree of oxidative damage to the plant cell [43]. The MDA content of the RI lines was clearly higher than that of wild-type (WT) plants under salt stress (Fig 7B). Consistent with higher MDA content, the chlorophyll content in the leaves of RI plants was severely reduced compared with WT plants under NaCl stress (Fig 7C). These results further suggest that OsVTC1-1 plays an important role in scavenging ROS under salt stress.

## Discussion

Salinity stress has an adverse effect on rice at all stages of growth [44–46]. Reproductive stage is a key period for growth and yield of rice under salt stress [45, 46]. Several studies have shown that GMPase has an important effect on tolerance of plants to salinity stress at vegetative stage, but the role of GMPase in tolerance of plants to salt stress at reproductive stage remains unclear [9, 19, 29]. Results of present study showed that GMPase OsVTC1-1 plays a critical role in salt tolerance of rice at both seedling and reproductive stages through regulating AsA biosynthesis.

Previous studies have demonstrated that GMPase is involved in response to salt stress at seedling stage [8, 9, 19, 29, 33]. A point mutation of *Arabidopsis* GMPase VTC1 dramatically reduces tolerance to salt stress [19]. In contrast, stable VTC1 protein in *csn5b*, a mutant of COP9 complex subunit CSN5B, significantly improved tolerance of *Arabidopsis* to salt stress [33]. Rice is an important crop species. Salinity affects rice growth in varying degrees at different development stages. Seedling and reproduction are two most sensitive stages of rice to salinity stress [36, 44–46]. Excess salt limits seedling growth and reduces tiller numbers at seedling stage, and reduces grain numbers per panicle and diminishes filled grain rate at reproductive stage, which causes severely loss of rice yield [34, 37, 44–46]. The expression of rice GMPase gene *GMP1* (*OsVTC1-1*) is induced under salt stress ([29], Fig 1), and overexpression of *OsVTC1-1* enhances salt tolerance of transgenic tobacco plants [29], indicating that expression of *OsVTC1-1* is important to enhance salt tolerance of plants. In this study, knockdowning *OsVTC1-1* expression impaired seedling salt tolerance of different rice varieties, including salt-tolerant rice varieties (Figs 2 and 3), suggesting that *OsVTC1-1* also plays an important role in salt tolerance at vegetative stage in rice. Intriguingly, under salt stress, *OsVTC1-1* RI lines showed fewer panicles, less grain numbers per panicle, and reduced grain yields compared with control rice (Fig 4), indicating that OsVTC1-1 also plays a key role in tolerance of rice to salt stress at reproductive stage. Thus, taken together, these studies reveal that GMPase plays a critical role in salt tolerance of rice at both seedling and reproductive stages.

High salinity disrupts ion and water equilibrium of plant cells, which accumulate large amounts of ROS, further impairing the activity and stability of plant proteins and resulting in serious harm to plants [42, 47–51]. For example, high salinity limits the regeneration of NADP^+^ in the Calvin cycle, which further damages the components of photosynthetic electron transport chain, resulting in the termination of photosynthesis due to the accumulation of ROS in plant cells under salt stress [50]. Therefore, plants need to efficiently scavenge excess ROS to protect chlorophyll and chloroplasts from damage under high salinity conditions [51]. Plants have evolved numerous mechanisms to avoid cellular damage due to ROS accumulation, including synthesis of anti-oxidants under high salinity conditions [19, 25, 27, 33–35, 49, 52–54]. In salinity stress, AsA might act as a primary antioxidant to directly scavenge ROS and indirectly clear ROS through AsA-GSH (glutathione) cycle [26, 27, 54]. Therefore, the regulation of AsA synthesis plays an important role in salt stress response [19, 33, 34, 38, 39].

In plants, AsA is synthesized through several pathways [55–57]. Among these pathways, L-galactose pathway plays a major role in plant AsA biosynthesis [26]. The biosynthesis of AsA through L-galactose pathway involves at least ten enzymes. Among these enzymes, GDP-mannose pyrophosphorylase (GMP) [2, 58], GDP-mannose-3',5'-epimerase (GME) [56], GDP-L-galactose phosphorylase (GGP) [59, 60], L-galactose-1-phosphate phosphatase (GPP) [61], L-galactose dehydrogenase (GDH) [62], and L-galactono-1,4-lactone dehydrogenase (GalLDH) [63, 64], are important enzymes that regulate AsA biosynthesis in plants. GMPase is a key enzyme of AsA biosynthesis and plays multiple important roles in the responses of plants to stresses [12, 8, 20, 26]. Previous studies have implicated GMPase in ROS scavenging in the response to salt stress in dicot plants *Arabidopsis* and tobacco [12, 19]. Previous study has showed that OsVTC1-1 is a key GMPase for AsA biosynthesis in rice leaves. AsA content in *OsVTC1-1* RI line RI-3 was approximately 40% of that in wild-type plants [28]. In present study, application of exogenous AsA restored the growth of *OsVTC1-1* RI seedlings to a level similar to that of wild-type seedlings under salt stress (Fig 6). *Arabidopsis* GMase mutant *vtc1-1* reduces ability for ROS scavenging and salt tolerance under salt stress [2, 19]. The expression of *OsVTC1-1* restored AsA content and salt tolerance of *vtc1-1* ([28], Fig 5), and similar to *vtc1-1*, *OsVTC1-1* RI plants also accumulated more ROS (O_2_^-^) and MDA than wild-type plants under salt stress ([19, 65], Fig 7), indicating that the decreased tolerance of *OsVTC1-1* RI plants to salt stress might reflect impaired AsA synthesis for scavenging excess ROS induced under high salt conditions.

## Supporting Information

S1 Fig Salt tolerance of rice varieties. (A), phenotypes of different rice varieties under salt stress. Control indicates that plants were grown under normal conditions; NaCl indicates that two-week rice seedlings were treated with 150 mM NaCl for 10 days. (B), the percentage survival rates of rice varieties after salt stress treatment in (A) with another ten days of re-watering without NaCl. NJ16, ZH17, Nip, Kos, YD6 and IR29 indicate rice varieties Ningjing16, Zhonghua17, Nipponbare, Koshihikari, Yangdao 6 and IR29, respectively. The bars represent SD (±) of three independent assays. About 50~60 seedlings were uses in each experiment.
(EPS)

S2 Fig The expression level of *OsVTC1-1* and AsA content in *OsVTC1-1* RI1-3-hybrid rice plants. (A), total RNA was extracted from leaves of four-week F1 *OsVTC1-1* RI1-3 hybrid rice plants; subsequently, the expression of *OsVTC1-1* was analyzed using qPCR. (B), AsA content of F1 *OsVTC1-1* RI1-3 hybrid rice plants. NJ16 represents salt-tolerant rice landrace Ningjing16; NJ16/RI1-3 represents F1 hybrid of Ningjing16 with *OsVTC1-1* RNA interference line RI1-3; and NJ16/ZH17 represents F1 plants of Ningjing16 and Zhonghua17, which were used as controls. ZD13 indicates salt-tolerant rice landrace Zhongdao13; ZD13/RI1-3 indicates F1 plants of Zhongdao13 and *OsVTC1-1* RI1-3; and ZD13/ZH17 indicates F1 plants of Zhongdao13 and Zhonghua17, which were used as controls. Ningjing16 and Zhongdao13 were used as female parents. The bars represent SD (±) of three independent assays, and the asterisk indicates results significantly different from control (NJ16/ZH17 and ZD13/ZH17, respectively) (**P<0.01). Significance was evaluated using the *t*-test.
(EPS)
